# Novel rGO-T-C(n) Nanosheets developed via click chemistry as a lubricant anti-wear additive

**DOI:** 10.1038/s41598-018-23898-y

**Published:** 2018-04-18

**Authors:** Samira Bagheri, Nadia Jamal, Ahmed Halilu, Amin TermehYousefi

**Affiliations:** 1 Department of Mechanical Engineering, Tufts University, Medford, MA 02155 U.S.A.; 2 Nanotechnology and Catalysis Research Centre (NANOCAT), University of Malaya, 50603 Kuala Lumpur, Malaysia; 3 Department of Petrochemicals and Allied, National Research Institute for Chemical Technology (NARICT), P.M.B, 1052 Zaria, Nigeria

## Abstract

Process equipment and facilities are constantly facing the dilemmas of tear and wear. This manuscript introducing functionalized reduced graphene oxide with triazole moiety via click chemistry as a anti-wear additive. While this has been achieved successfully, full characterization of the new anti-wear additive material revealed it to be promising in ameliorating issues of wears. One of the merits of the synthesized material includes reduction of contact asperity as the lipophilic alkyl chain length increases. It has been tested to be functional when formulated as an additive in group III petroleum base oil. Accordingly, it shows an irregularity in renewable base oil. Following screening evaluations of the lipophilic alkyl chain lengths, the additive with twelve carbon atoms; functionalized reduced graphene oxide, rGO-T-C(12) was confirmed to stand out among others with the good reduction of friction coefficient and the least wear scar diameter of ~539.78 µm, compared to the base oil containing no additive.

## Introduction

Increasing the lifespan of chemical process/energy facilities as well as engineering machinery is inevitably worthwhile. Conventionally, lubrication of these tools to reduce surface contact asperity is known and aided with eco-friendly additives; reduction of friction has been increasingly imminent. While there are substances that evolved to control artefact of high surface friction, they pose many issues. For instance, the liquid lubricants irrespective of its attributes of forming low shear and high durability boundary film has a downside of wearing out due to temperature sensitivity limitations and finite surface boundary thickness^1–5^. Molybdenum disulphide appears to solve these challenges because it forms strong films that are stable at 400 °C and harsh dry environment with high lubricity but could not stand moisture and air before losing its lubricity^6–9^. The issue of the oxidizing environment such as air seemingly emerged to be curtailed by graphite lubricant because it is stable at 450 °C continuous temperatures with effective lubrication effects but its functionality suffers without humidity^9^. Boron nitride as another lubricant with temperature stability up to 1200 °C, durable in an oxidizing atmosphere has less lubricating effects coming from its tight diamond-like lamellar structure that renders is supplementary to diamond in cutting hard substances^10^. Considering all these lubricants and additives, none has anti-corrosion attributes however, Zinc dialkyldithiophosphate could stand out with its antioxidant and anti-corrosion properties in addition to its lubricating effects but do decomposes to release poisonous phosphorus at 130–170 °C^11,12^. Because a chemically bonded lubricating film is also required in solving tear and wears issues Tricresyl phosphate is a good option considering its low activation temperature of >200 °C. This should be supposed to be celebrated unfortunately on health grounds when Tricresyl phosphate is ingested depression and schizophrenia are prevalent^13,14^.

Hallmark of this study goes add-on against the downsides of the anti-wear additives and lubricants. By carefully introducing lipophilicity and electron richness to reduced graphene oxide (rGO), the anti-wear additive material we develop emerges to tackle most of the issues mentioned in association to the conventional ones. It offers an exceptional additive performance not commonly seen in the conventional counterparts. Some of the material’s credit are its biocompatibility, extreme strength, eco-friendly, shear capability in either the basal or edge planes. These consider it fit for impressive tribological trends as additive material^15–18^. Also rGO intercalating properties and its tendency of forming precipitates make it agglomerate in water or organic solvents when employed as an oil additive^19^. Though rGO has been shown to be self-lubricating in reducing surface friction and wears, unfortunately, it is evidently known with low dispersibility^20,21^. This could constitute financial loss and process inefficiency during solving issues of tear and wears of gliding surfaces. Hence, the need to functionalize rGO with lipophilic groups that would stay in the oil phase boosting dispersibility, and at the same time garnishing rGO with highly electron rich species favor the chemistry of metal surface contact to boost the tribological properties of tear and wear reduction for the anti-wear additive material. Achieving the hypothetical thought via click chemistry has been paramount towards industrial scalability of the process as confirmed from various state of the art analysis. What is practically motivating contemporarily for further research curiosity is the placement of the group on either rGO basal plane or edge site, though the contemporary analysis in this study strongly suggested edge site of rGO to be functionalized with the triazole moiety.

## Results and Discussion

### Confirming the click coupling in rGO-T-C(n) anti-wear additive

**Figure 1:**
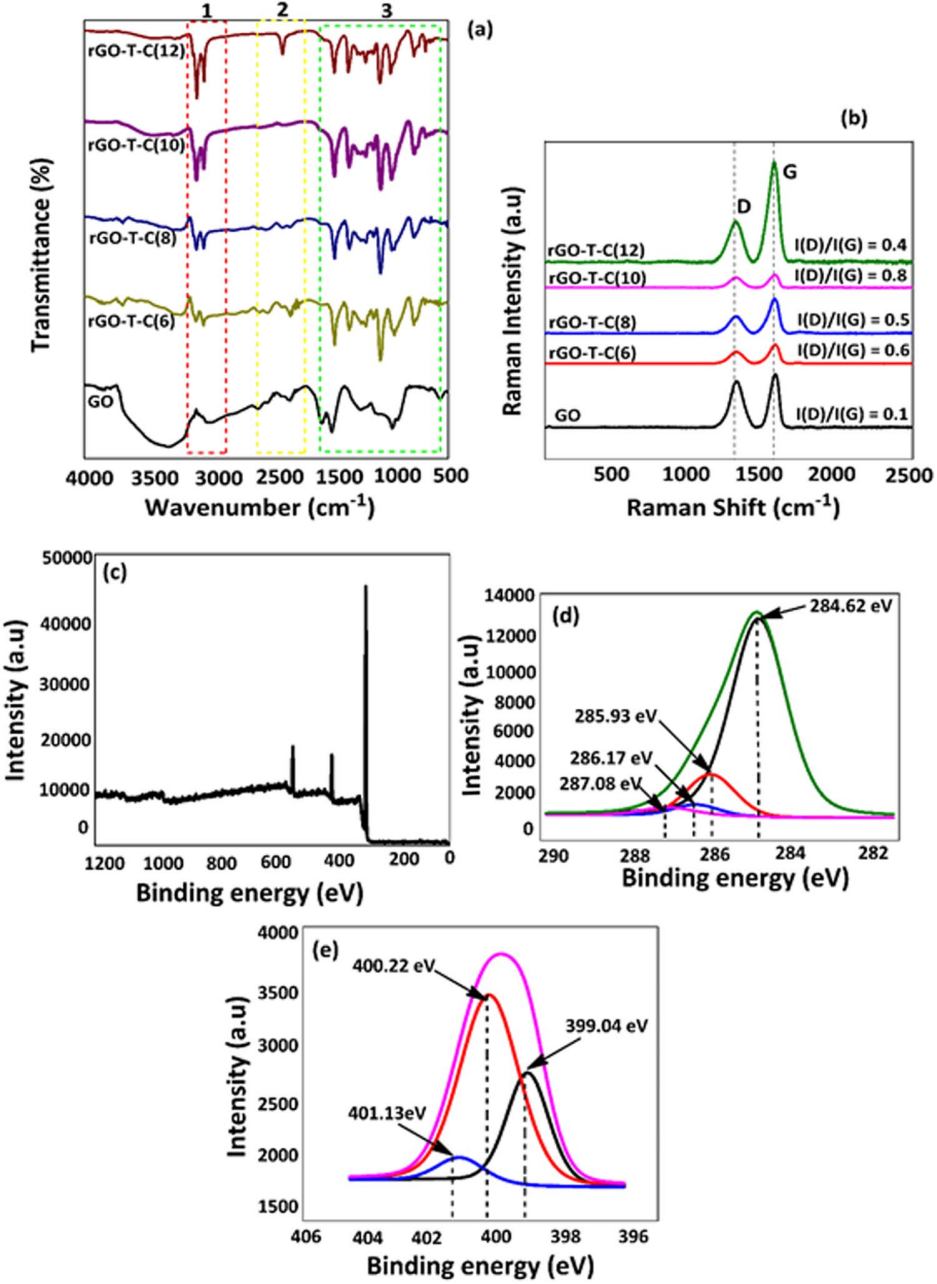
(**a**) FTIR spectra of graphene oxide (GO) and rGO-T-C(n) (n = 6, 8, 10 and 12). (**b**) Raman spectra of GO and rGO-T-C(n) (n = 6, 8, 10 and 12) (**c**) XPS spectra of rGO-T-C(n) (n = 12) in the wide region; (**d**) XPS spectra of rGO-T-C(n) (n = 12) in the C_1s_ region (**e**) XPS spectra of rGO-T-C(n) (n = 12) in the N_1s_ region.

X-ray photoelectric emission (XPS), Fourier-transform infrared spectroscopy (FTIR), and Raman were employed to confirm click chemistry of the rGO-T-C(n) (n = 6, 8, 10, 12). As a prior data acquisition to this effect, Raman and FTIR for rGO-T-C(n) (n = 6, 8, 10, 12) were shown and XPS of the best anti-wear additive; rGO-T-C(12) was shown herewith. For the FTIR results in Fig. 1a, some modes representing the stretching of O-H, C=O, C=C, epoxy C-O and alkoxy C-O groups were visible at 3237, 1710, 1614, 1230 and 1044 cm^−1^ respectively and they are in accordance with the literature^22^. This evidenced the fact that rGO was the physical material we were using. The disappearance of C=O stretching modes at 1710 cm^−1^ for all the rGO-T-C(n) (n = 6, 8, 10 and 12) as in Fig. 1a indicates alkyl click coupling, evidenced previously^23^. Suggestion on stretching of C-H in -CH_2_ materialized from the peaks that appeared at ~2850 and ~2950 cm^−1^. Still on, the peak at 2098 cm^−1^ signifies click coupling of azide group to the F-rGO surface. Therefore, confirmation of azide-alkyne coupling was made from the disappearance of 2098 cm^−1^ peak and its reappearance at 1100 cm^−1^ for the stretching of C-O groups^24^.

While indications of various bonding vibrations in rGO-T-C(n) are seen in Fig. 1a, it’s profound to classify the carbon bonding types and nature of defects inquired in rGO-T-C(n) and rGO material. As a result of in-plane vibration of sp2 bonded carbon atoms in rGO-T-C(n) and rGO, G band was prevalent as seen in Fig. 1b. Likewise, as a result of out of plane vibrations of sp3 bonded carbon due to the presence of a defect, D band is prevalent in rGO-T-C(n) and rGO^22,24^. Synoptically, rGO-T-C(n) and rGO are all characterized by the two D and G band peaks centered at ~1340 cm^−1^ and ~1590 cm^−1^ respectively. It is certain from the D-band that there is a presence of defects in either rGO or rGO-T-C(n). The defect present in rGO-T-C(12) is lower than those in rGO-T-C(6), rGO-T-C(8) and rGO-T-C(10); though there is no definite correlation between the increase in a number of carbon chain length with the amount of defect present. This deduction was evidenced from the fact that rGO-T-C(12) has I(D)/I(G) = ~0.40, which is less than rGO-T-C10 (I(D)/I(G) = 0.81), rGO-T-C(8) (I(D)/I(G) = 0.50), rGO-T-C(6) (I(D)/I(G) = ~0.6). Therefore, because rGO reveals the least defect with I(D)/I(G) = ~0.1, it is logical to suggest a most probable click coupling from the edge site of rGO for rGO-T-C(12). This is also supported by the fact that defects do occur from the basal plane of rGO. Figure 1c,d and e show the XPS for the surface analysis of rGO-T-C(n) anti-wear additive. The XPS analysis confirmed the two facets of the anti-wear additive material; rGO at C_1s_ (284 eV) peak in Fig. 1d, and click coupling of triazole moiety at N_1s_ (400.2 eV) peak in Fig. 1e. Focusing on rGO, either sp^2^ or sp^3^ C-C carbon was evidenced from the peak centered at 284.7 eV, and the peak at 285.9 eV signifies C-O bond of a hydroxyl group. The peak that centered at 286.2 eV denotes C=O of a carboxyl group, and the peak at 287.1 eV embodies O-C=O group. These functionalities that were confirmed for rGO herewith are in accordance to the ones previously reported elsewhere^25^. Triazole moiety was also confirmed from the peak which centered at 405 eV. This also confirmed covalent attachment of the azide group to the rGO surface through the triazole ring^26^. Besides, the attachment of alkyne compound on rGO was confirmed from the peak centered at 399 eV. Re-focusing on detection of azide group residue by the FTIR measurement, we suggest that click coupling of alkyne functionalized rGO to azide compound did not yield 100% conversion.

### X-ray diffraction (XRD) analysis of rGO-T-C12 anti-wear additive

**Figure 2:**
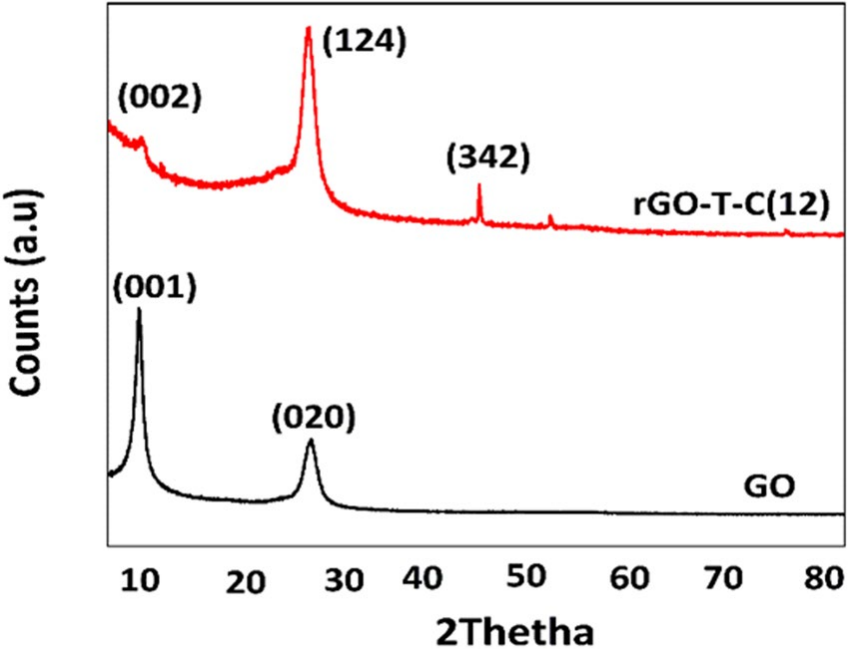
XRD diffraction peaks of rGO and rGO-T-C(12).

The rGO-T-C(12) anti-wear additive material was confirmed to be crystalline as shown in Fig. 2. It contrasts rGO that indicated two peaks at ~9.2**°** and 26.1°, corresponding to (001) and (022) crystallographic planes with d-spacing’s of 9.4367 Å and 3.4100 Å respectively^27^. After phase identification, rGO-T-C(12) also features two peaks at 9.858° (d-spacing = 9.3800 Å) and 25.974° (d-spacing = 3.44585 Å), corresponding to rGO phase confirming its presence in the whole material^28^. The presence of an additional unique peak at 43.349 ° (d-spacing = 2.08329 Å) on (342) crystallographic plane is postulated to be due to the presence of triazole moiety containing alkyl functionality. This suggestively amounts to the blue shift of the diffraction angle as well as a change in the crystallographic plane to which the rGO phase lies on rGO-T-C(12).

### Thermal stability analysis of rGO-T-C(n)

**Figure 3:**
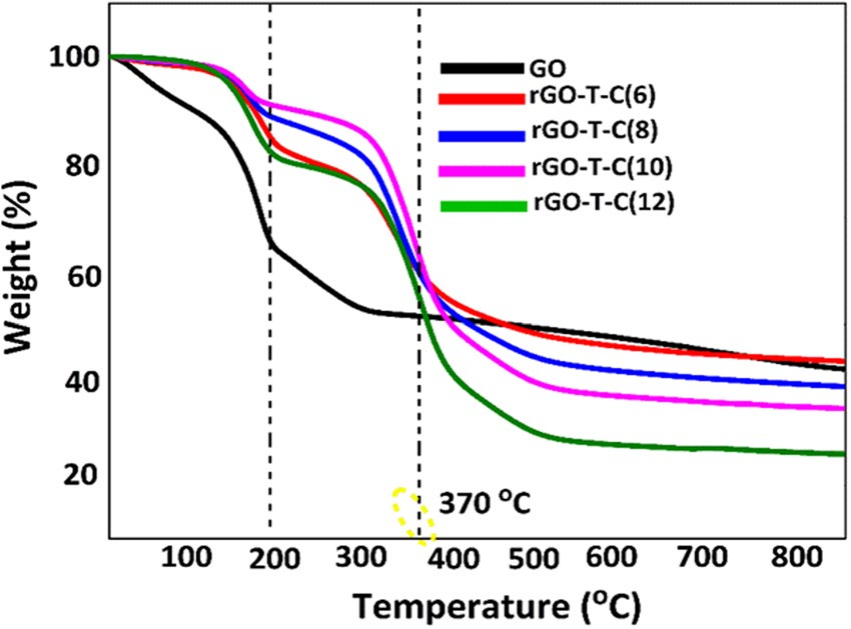
The TGA curve of F-rGO-C(n) (n = 6, 8, 10, 12).

Figure 3 shows three stages of weight loss during the thermogravimetric analysis (TGA) analysis of rGO-T-C**(**n**)**. Between 50–150 °C interval the ~3% weight loss was not much significant due to their hydrophobicity. Significant weight loss was only recorded between 150 to 300 °C and are attributed to oxygen-based functional groups such as carbonyl, epoxy and hydroxyl groups^29^. These oxygenated functionalities decompose between the temperature of 300 to 460 °C. Interestingly, it is suggestive to say that grafting of the alkyl functionality was successful on rGO-T-C**(**n**)** owing to the significantly smaller amount of decomposition observed within the temperature range of which free surface functional group were known to decompose^24^. The nitrogen group such as the triazole moiety decomposing at ≤370 °C marks the temperature datum for rGO-T-C**(**n**)** stability before it begins to lose its performance as an anti-wear additive when a gliding surface generates heat that is equivalent to 370 °C.

### Purity and Morphology of rGO-T-C(n) anti-wear additive

**Figure 4:**
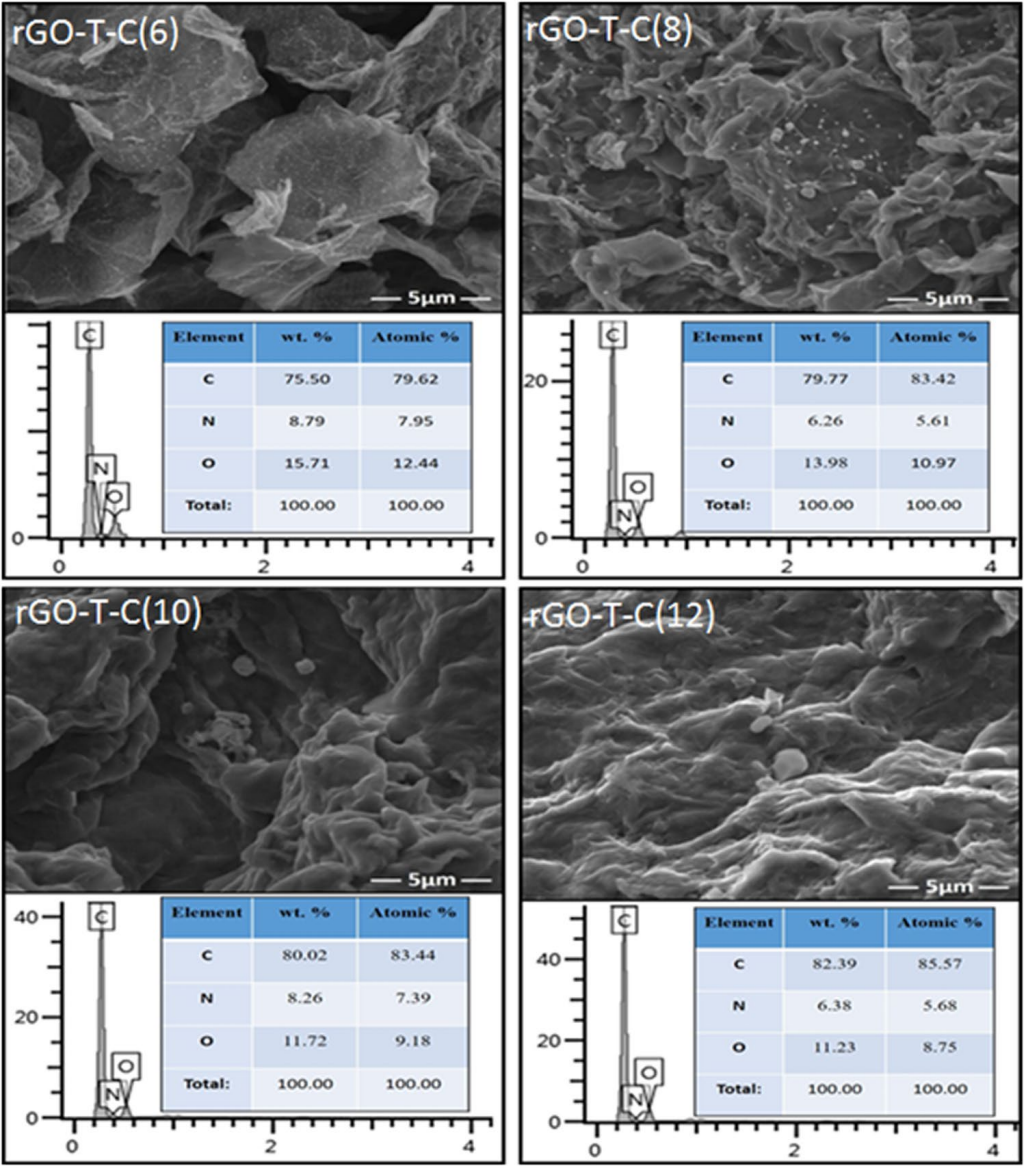
Field-emission scanning electron microscopy (FESEM)/EDX images of rGO-T-C(n) (n = 6, 8, 10, 12) with different probed spectrum.

The purity of rGO-T-C(n) anti-wear additives were ascertained via energy dispersive X-ray (EDX) probing of at most five regions on each additive surface as shown in Fig. 4. All the rGO-T-C(n) (n = 6, 8, 10 and 12) samples contain C, and two-point charges; N and O as their only atomic composition. Details on the EDX spectra and the percentages of C, N, and O for the respective regions can be seen in supporting information file S1. Hence the absence of Cu confirmed the successful elimination of the catalyst used during this modified Hummer’s method. This provides further clearance to the industrial scalability of the method. On the morphology of rGO-T-C(n), the same Fig. 4 shows their morphologies in form of flakes and this indicates that the exfoliation of graphite was successful^30^. However, the wrinkles and folds observed on the surface of rGO-T-C(n) suggest the presence of the alkyl functional groups which distorts the dense sheet found in the original structure of the GO. We can hypothetically postulate that this alkyl groups could act as a spacer that potentially holds two sheets from the basal plane to the edge or edge to edge hence the folds.

### Dispersibility test

**Figure 5:**
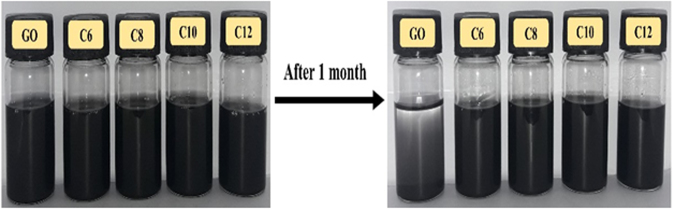
GO and rGO-T-C(n) dispersed in oil (left), after one month (right).

The digital pictures as illustrated in Fig. 5 were taken to evidence good dispersibility of rGO-T-C(n) and poor dispersibility of rGO in oil. This was achieved right after sonicating rGO-T-C(n) and rGO in group III base oil until they are fully dispersed with no residual solids remaining. To confirm and identify the degree of sedimentation, pictures were taken again after a month. At the onset, rGO-T-C(n) and rGO were easily dispersed in the base oil immediately after sonication. After a month of isolation, all rGO-T-C(n) in base oil did not sediment to the bottom due to the presence of the lipophilic effects of the alkyl moiety coupled with the triazole moiety. This is also aided by the crumpled structure of rGO-T-C(n) which prevents the formation of tight stacking that prevents aggregation^31^. On the contrary, rGO which has no lipophilic moiety functionalized on it begins to sediments even prior to the 30 days period of isolation and this proved that the dispersibility of the rGO-T-C(n) in the group III base oil has improved, upon functionalization^32^.

### rGO-T-C(n) and its boundary lubrication effect

**Figure 6:**
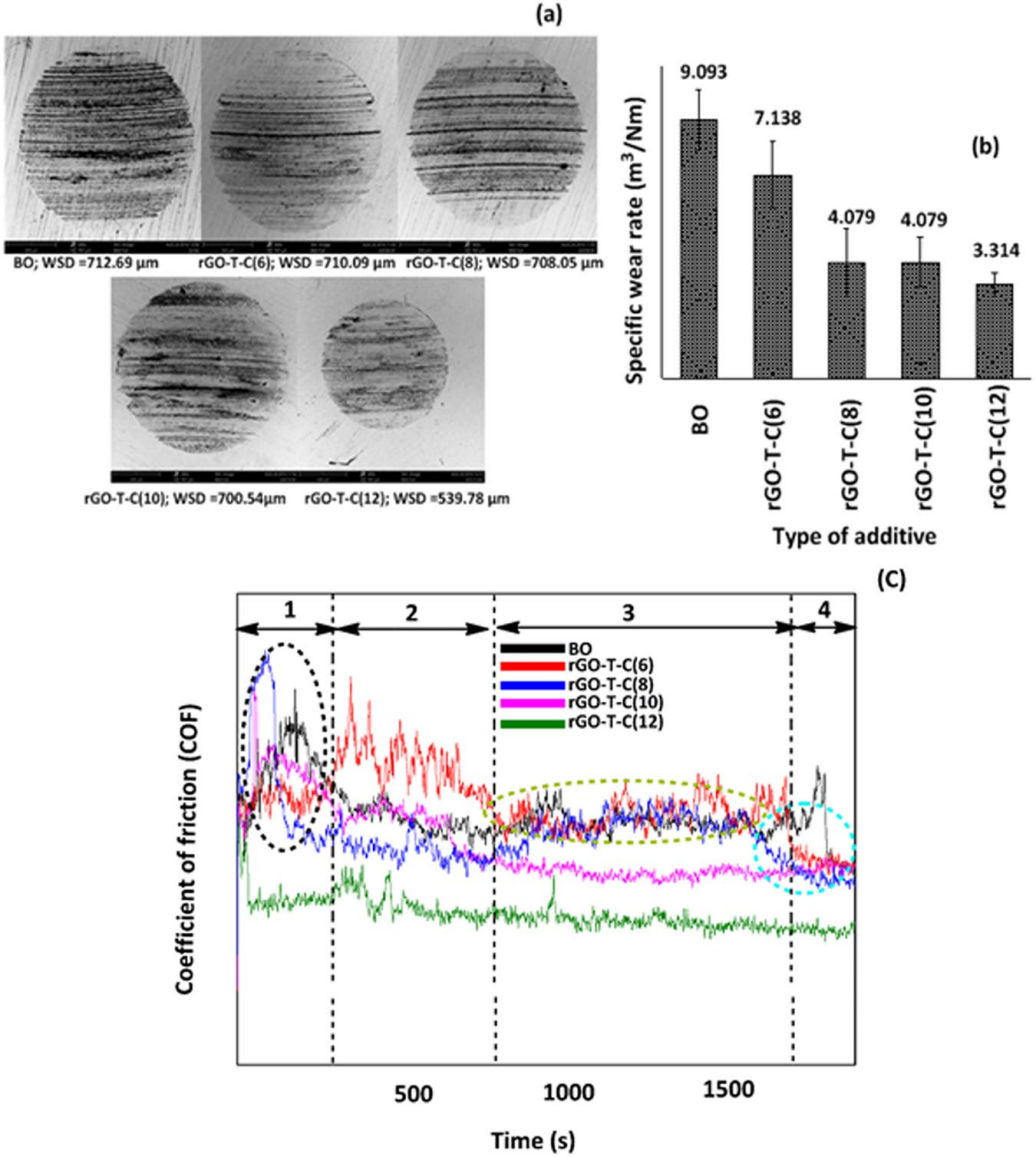
Tribology on BO and formulated BO with rGO-T-C(n) (n = 6, 8, 10, 12). (**a**) Wear scar diameter (WSD). (**b**) Specific wear rate. (**c**) Coefficient of friction.

The performance of the rGO-T-C(n) (n = 6, 8, 10 and 12) antiwear additives in type III base oil (BO) were evaluated as shown in Fig. 6(a),(b) and (c), via the four-ball test analysis. The wear scar diameter (WSD) for base oil formulated with rGO-T-C(n) (n = 6, 8, 10,12) antiwear additive reveal that rGO-T-C(12) is more effective (Fig. 6a). Though all the additives show specific wear rate in 10^−6^ magnitude which confirmed that the base oil formulated with rGO-T-C(n) antiwear additive material has a boundary lubrication effects^33,34^. Other lubrication effects are hydrodynamic wear rate which ranges from 10^−10^ to 10^−14^, boundary lubrication 10^−6^ to 10^−9^, adhesive wear when there is no lubrication condition 10^−2^ to 10^−7^, abrasive wear for unlubricated condition 10^−1^ to 10^−5 ^^35–37^. Intrestingly the wear scares diameter (WSD) decreases with increasing alkyl carbon number C(n) (n = 6, 8, 10, 12) in the rGO-T-C(n) antiwear additive. Ordinary G-III base oil without additive showed WSD of 712.69 µm and when rGO-T-C(6) additive was introduced, the WSD reduced to 710.09 µm with a difference of ~2.6 µm from the previous. Similarly, rGO-T-C8 has WSD of 708.05 µm which is ~2.04 µm different relative to rGO-T-C(6). Also, the WSD for rGO-T-C10 is 700.54 µm and this is ~7.51 µm less than rGO-T-C(8). The WSD of rGO-T-C(12) is 539.78 µm and this is ~160.76 µm less than rGO-T-C(10), 168.27 µm less than rGO-T-C(8), 170.31 µm less than rGO-T-C(6) and 172.91 µm less than when ordinary BO was used. These results confirmed rGO-T**-**C(12) to form protective coating making boundary lubrication prevalent hence, improving the tribological trends of the formulated base oil.

In Fig. 6b unformulated base oil (BO) with additive showed the highest specific wear rate of 9.093 m^3^/Nm unlike rGO-T-C(12), which has the lowest specific wear rate value of 3.314 m^3^/Nm. Generally, we observed a trend that as the carbon chain length, C(n) increases, the specific wear rate decreases in spite the fact that C(8) and C(10) recorded the same value. After the N = N site in the triazole moiety in rGO-T-C(n) chelate with the metal surface, the -R lipophilic group begin to coordinate with the base oil molecule likewise. The -R lipophilicity becomes more profound when the carbon chain length increases because the tendency of not forming hydrogen bondis increasing. Consequently, this implicates the trend we observed for the specific wear rate values.

Within 1800 seconds of operation as in Fig. 6c, and from the coefficient of friction (COF) profile with time, rGO-T-C**(**12**)** showed a superior and reasonable reduction in friction unlike rGO-T-C**(**6**)**, rGO-T-C**(**8**)**, and rGO-T-C**(**10**)** additives. This indicates low contact asperity between the four balls gliding surfaces. Points 1, 2, 3 and 4 are informative to show the dynamism of the additive stability with time during the tear and wear experiments. rGO-T-C**(**12**)** was stable throughout, unlike other additives that struggle to maintain their stability during the 1800 seconds of operation. This is attributed to the fact that the lipophilicity of the C**(**12**)** chain length is more profound than C**(**10**)**, C(8) or C**(**6**)** and this is irrespective of the triazole moiety electron rich group. The region marked 1, between 0s to 285s in Fig. 6, distinctly reveal the onset of reduced COF for rGO-T-C**(**12**)** while others are erratic. In contrast, COF for rGO-T-C**(**6**)** drops within intervals of 285–732s in the region marked 2. On the other hand, rGO-T-C**(**10**)**, shows less significant activity and rGO-T-C**(**8**)** display less in reduced COF. However, within 732–1621s of operation, rGO-T-C**(**12**)** still maintain appreciable and high stability for reduced COF while rGO-T-C**(**6**)** and rGO-T-C**(**8**)** display no observable activity and rGO-T-C**(**10**)** reveals some reduction in COF but still less than rGO-T-C**(**12**)**. Surprisingly, at around 1621–1800s of operation, rGO-T-C**(**6**)**, rGO-T-C**(**8**)** and rGO-T-C**(**10**)** display similar observable activity in reducing COF less than rGO-T-C**(**12**)**.

### Critical insights on a proposed mechanism on wear reduction in base oil formulated with rGO-T-C(n) anti-wear additive

**Figure 7:**
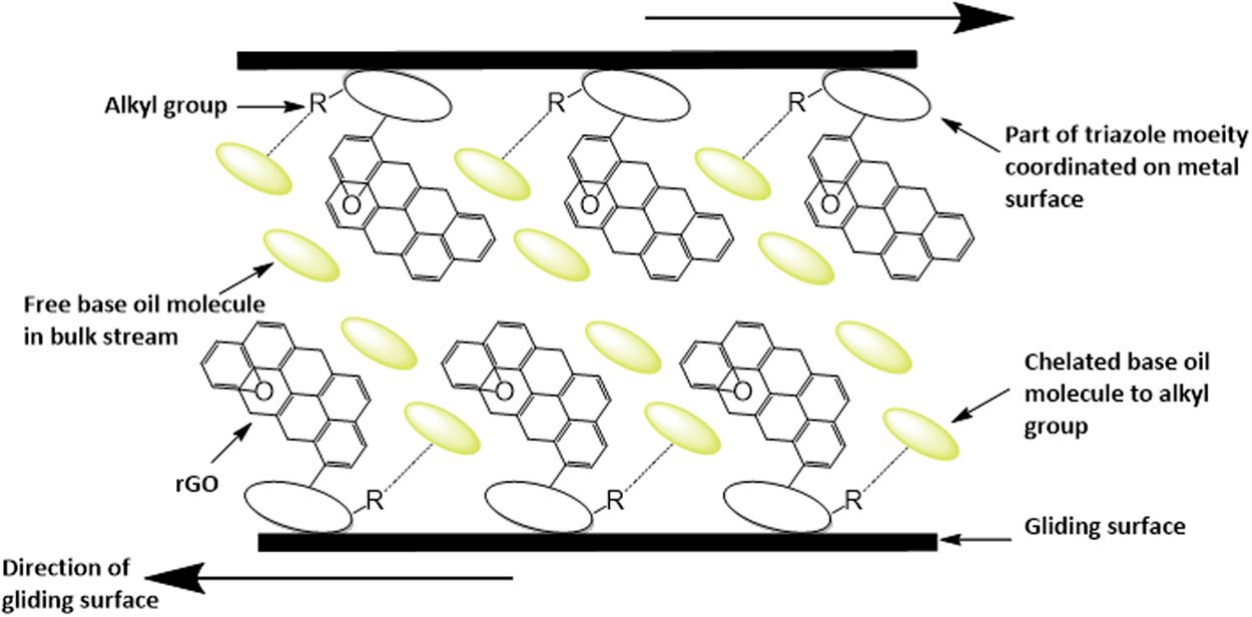
Proposed mechanism for the formulated base oil with rGO-T-Cn (n = 6, 8, 10, and 12) as anti-wear additive.

Previously from the thermogravimetric analysis, the triazole moiety is durable until 370 °C when it begins to decompose. This implies at a temperature <370 °C or possibly >100 °C, perturbing triazole moiety electronic structure becomes significant to implicate its chelation with metal surfaces. This chelation could be through either of its four sites that poses fascinating coordinating affinities; in this postulation, a focus is made on R and R’ alkyl groups sites that are responsible for chelation, and N = N bonding site potent for metal coordination^38,39^. We postulate that during operation, rGO-T-C(n) anti-wear additives in the formulated base oil forms a thin boundary layer with the surface of the metal in contact. This is possible because Triazole moiety is a strong ligand to metal ions (M^+n^; Cu^2+^, Fe^2+^, Pt^3+^, Pd^2+^, Ru^3+^ and Ag^+^); most metal surfaces are in the oxide states that implicate the possibility of M^+n^ presence (where n ≠ 0)^40–43^. Accordingly, rGO-T-C(n) in base oil serves as anti-wear additive via coordination of the N = N triazole moiety site with the metal surface through possible activation at >100 °C while its lipophilic R-group undergoes chelation with the base oil molecule. Consequently, the gliding occurs between chelated base oil molecule to an alkyl group, rGO, and some free base oil in bulk stream to create a non-laminar boundary layer on the surface of the metal. Figure 7 shows the proposed mechanism for the formulated base oil with rGO-T-Cn (n = 6, 8, 10, and 12) as anti-wear additive,

## Methods

### Synthesis of rGO-T-Cn anti-wear additive

**Figure 8:**
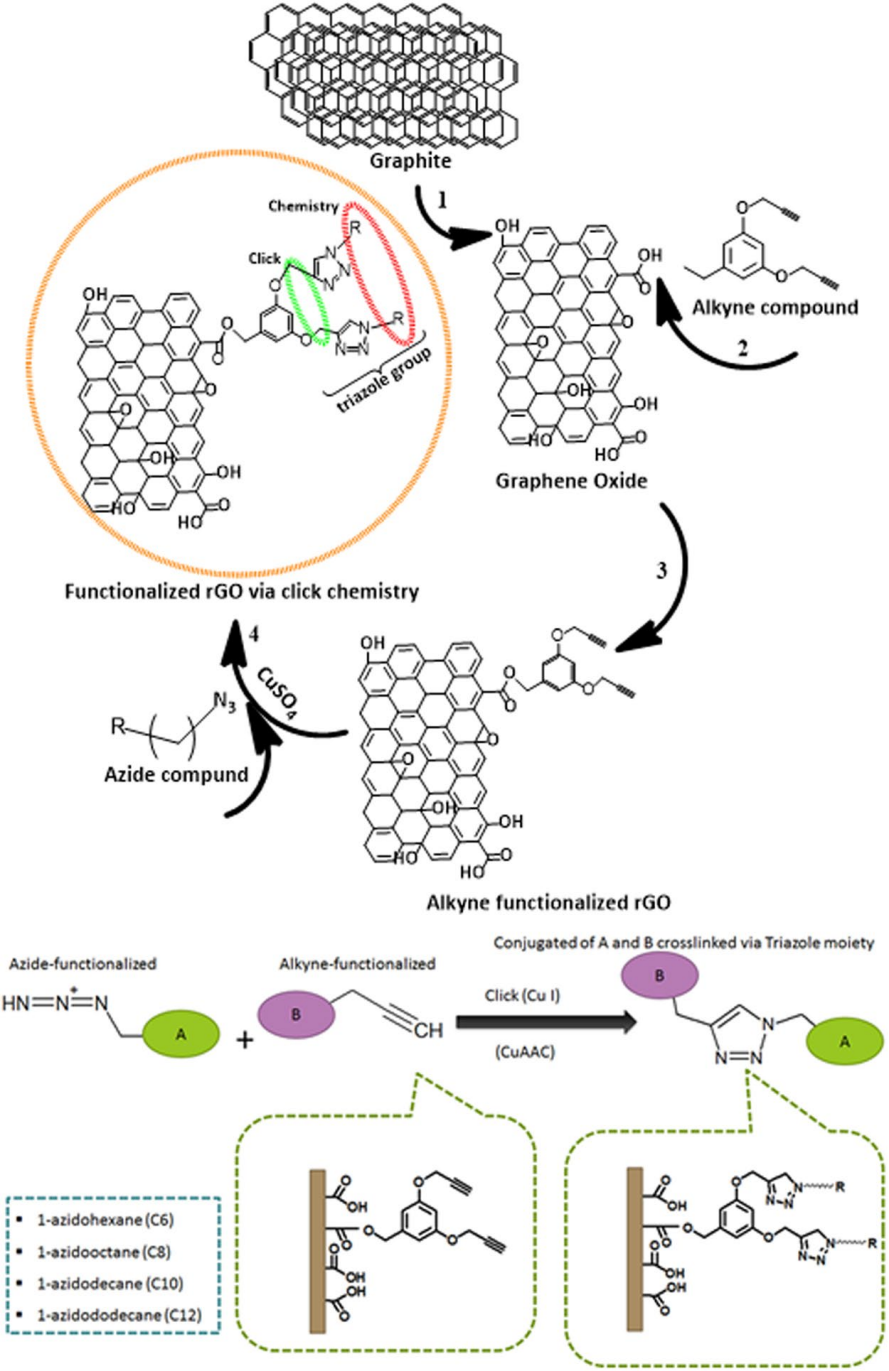
Synthesis of rGO-T-Cn anti-wear additive via click chemistry.

The rGO-T-Cn anti-wear additives wear synthesized through three steps; rGO preparation, alkyne group preparation, then coupling with rGO, then synthesis of azide group and click coupling with alkyne functionalized rGO. rGO was obtained from graphite flakes exfoliation using modified Hummer’s method. The mixture of H_2_SO_4_ (120 mL) with H_3_PO_4_ (13 mL) was introduced to ~1 g of graphite and stirred constantly for 10 min to produce layers of graphene sheets. The graphene sheets were oxidized with KMnO_4_ (6.0 g) at less than 10 °C and stirred for 1 h. This suspension was treated with H_2_O_2_ (30%, 20 mL) to ensure complete oxidation process. In order to remove Mn^2+^ ions in GO, the mixture was centrifuged and washed using HCl deionized water through filtration and drying^44^. Then alkyne compound was synthesized by dissolving ~3.0 g of Methyl-3,5-dihydroxybenzoate in acetonitrile and gradually introducing ~7.4 g of potassium carbonate, 4 mL of propargyl bromide (80 wt.% in toluene) then refluxed at ~80 °C for 24 h. It was thereafter filtered then evaporated giving yellow flakes. About ~0.9 g of LiAlH_4_ was added to the flake (3.0 g) in dry THF and stirred for 24 h. LiAlH_4_ in the mixture was quenched by water drop wisely then filtered and dried over anhydrous MgSO_4_ yielding alkyne compound. Functionalized GO was then chlorinated by stirring ~1 g of GO in chloroform and addition of thionyl chloride with triethyl amine for 2 h. it was evaporated to yield chlorinated GO (rGO). Alkyne functionalized GO was achieved herewith by mixing ~1 g alkyne compound with ~1 g chlorinated GO and ~0.13 g of Sodium hydride in THF at ~5 °C for 24 h. The azide compound was then synthesized using ~4 g sodium azide with ~4.2 mL 1- bromohexane and refluxed in DMF at 80 °C for 24 h. Using diethyl ether, the reaction mixture was extracted and the organic layer was washed with water then dried over anhydrous MgSO_4_ and evaporated. For the click coupling reaction using ~0.01 g alkyne functionalized rGO, 0.2 g copper II sulphate a used as a catalyst. These combinations were continuously stirred overnight to dissolve them with ~0.02 g 1-azido hexane in DMSO and distilled water. At this stage, the click couple sample was thoroughly washed to remove the catalyst then it was also filtered with distilled water then ethanol and dried under vacuum for 24 h^24^. The anti-wear additive was designated as rGO-T-Cn where T stands for the triazole group with C being the alkyl chain lengths where n ranges from 6, 8, 10 and 12. Figure 8 illustrates the synthesis of rGO-T-Cn anti-wear additive via click chemistry.

## Conclusion

In summary, an industrial scalable route used to functionalize reduced graphene oxide for anti-wear additive via click chemistry. This additive nanostructure is effective when formulated with group III petroleum base oil, as well as the renewable base oil. It is observed that reduction in contact stresses is a linear function of the alkyl chain length incorporated to the rGO. This implicated the fact that higher chain length; C(12) recorded the lowest friction and wear by ~24%. To provide effective lubrication effects, rGO-T-C(12) activity was postulated to proceed via chelation of the R-group to the base oil, coordination of N = N site on the metal surface and collectively these creates boundary lubrication that yields a non-laminar boundary layer. The results clearly prove that functionalized reduced graphene oxide sheets in oil easily form protective films to prevent the rubbing surfaces from coming into direct contact and, thereby, improve the entirely tribological behavior of the oil.

## Electronic supplementary material

Supplementary Information
