# Supplementary material for: Novel rGO-T-C(n) Nanosheets developed via click chemistry as a lubricant anti-wear additive
Source: Sci Rep. 2018 Apr 18;8:6221. doi: 10.1038/s41598-018-23898-y (PMC5906662; doi:10.1038/s41598-018-23898-y)
Supplement: Supplementary file 1 — Supplementary Information [file 41598_2018_23898_MOESM1_ESM.docx]

Novel rGO-T-C(n) Nanosheets developed via click chemistry as a lubricant anti-wear additive

Samira Bagheri^a*^, Nadia Jamal^b^, Ahmed Halilu^bc^, Amin TermehYousefi^a^

**S.1 CHARACTERIZATION**

## S.1.1 Fourier Transform Infrared (FTIR) Spectroscopy

In this study, Perkin Elmer FTIR spectroscopy was used to identify the rGO-T-Cn functional groups among 400 to 4000 cm ^-1^ for each set of samples. The samples were prepared using the potassium bromide (KBr) method, where 0.03 mg of rGO-T-Cn was mixed with 4 mg of KBr. The trituration of KBr helps prevent light scattering due to the large size of the KBr crystals. The mixture was then formed into a pellet, which was then held in place by a sample holder, which enables the uninterrupted passage of the IR beam.

### S.1.2 Raman Spectroscopy

The Raman spectra were obtained using a Renishaw inVia Raman microscope, with a 514nm laser as its excitation source within the range of 100 - 3000 cm^-1^. To obtain the rGO-T-C(n) spectra, 0.05 g of F-rGO was tested using a 0.02 mV laser power and 180 seconds of exposure time. The obtained Raman spectra were analyzed to determine the phases of the rGO-T-C(n).

### S.1.3 X-ray Photoelectron Spectroscopy (XPS)

X-ray photoelectron spectroscopy provides an in-depth information of the chemical state of elements found on the surface of rGO-T-Cn. This pointed to the specific bonding of the elements present on the surface of the said material. The analysis is carried out at an average depth of 5 nm by using the ULVAC-PHI Quantera II, with a 32-channel spherical capacitor, analyses the energy re-emitted from the material after bombardment by X-ray under vacuum condition (1 ×10^-6^ Pa). The process also utilizes hemispherical analyzer with a natural width of 680 meV and monochromatic Al K_α_ sources (1,486.6 eV).

### S.1.4 X-Ray Diffraction (XRD) Analysis

The crystallographic structure and phase of the rGO-T-Cn were investigated using X-ray diffraction (XRD) analysis. In this research, the XRD diffractograms of the solid powder were analyzed using Bruker D8 Advance diffractometer, equipped with a quartz monochromatized Cu K_α_ radiation and wavelength, $\lambda=0.154059 nm$. Approximate 1 g of the sample was measured and placed into the sample holder. The system operated at a scanning rate of 0.02⁰ per second to over a 2𝜃 range of 10⁰ to 80⁰. Other frequent applications of XRD is to determine the crystallite size.

### S.1.5 Thermogravimetric Analysis (TGA)

TGA elucidates the composition of rGO-T-Cn and thermal stability as a function of temperature and time. It records the change in the weight of the rGO-T-Cn with increasing temperature and time under the flow of Argon gas. For compositional analysis, TGA helps estimate the amount of each component in the material by calculating the onset temperature of degradation or volatilization. In this work, TGA measurement was performed using a Mettler Toledo, TGA/SDTA-851^e^ that operates over the temperature range of 20 - 1000 °C and a scan rate of 20 °C/min. The sample was in the powder form, as it has a larger surface area, which helps improve the weight loss resolution and temperature reproducibility.

### S.1.6 Field Emission Scanning Electron Microscopy (FESEM)

The surface features and particles sizes or shapes of the sample were analyzed using a high-resolution field emission scanning electron microscope. The process begins with the irradiation of the samples with electrons and the reflected energy is used to image the surface at a microscopic level. In this research, the morphology of the rGO-T-Cn was determined using a scanning electron microscope Quanta FEG 450. The field emission gun electron source of this model produces a high current, which enables imaging up to 3 nm resolution and a magnification of up to 250,000 ×. All the tested samples were in the powder form.

**S.2 PERFORMANCE EVALUATION**

**S.2.1 Tribological test**. The low contact asperity effects of rGO-T-Cn antiwear additive in group III base oil was investigated with the four-ball wear tester. While maintaining a 1200 rpm, four stainless steel ball bearings with a diameter of 27mm each was subjected to a fixed 40kg load at 35 ^o^C. The experiment was conducted three times and average readings were measured every 30s. Accordingly, a probe microscope snaps the surface wear marks on the stainless steel ball bearings and instantly being measured by the grinding spot measurement software. Also, weight loss for rGO-T-Cn (n=6, 8, 10 and 12) was measured using a highly precise digital weighing machine. These weights of the test samples were converted to volume loss and the specific wear rates were determined using the Archard equation.

**Ws =** $\boldsymbol{\Delta VL}\boldsymbol{\bullet d}$

Where;

| Ws | = | specific wear rate (m^3^/Nm), |
| --- | --- | --- |
| $\Delta V$ | = | loss of volume (m^3^), |
| L | = | normal load (N) and |
| d | = | distance during sliding motion (m). |

**S.3 EDX AND FESEM ANALYSIS**

**S.3.1 Wt% of rGO-T-C(6)**

| Element | Spectrum 1 | Spectrum 2 | Spectrum 3 | Spectrum 4 | Spectrum 5 | Average |
| --- | --- | --- | --- | --- | --- | --- |
| C | 77.43 | 78.90 | 75.50 | 70.88 | 71.05 | 74.75 |
| N | 3.72 | 0.70 | 8.79 | 0.00 | 13.29 | 5.30 |
| O | 18.85 | 20.40 | 15.71 | 29.12 | 15.66 | 19.95 |
| Total: | 100.00 | 100.00 | 100.00 | 100.00 | 100.00 | 100.00 |

**S.3.2 Wt% of rGO-T-C(8)**

| Element | Spectrum 1 | Spectrum 2 | Spectrum 3 | Spectrum 4 | Spectrum 5 | Average |
| --- | --- | --- | --- | --- | --- | --- |
| C | 75.43 | 97.85 | 82.40 | 79.77 | 77.85 | 82.66 |
| N | 7.44 | 0.37 | 5.16 | 6.26 | 8.85 | 5.62 |
| O | 17.13 | 1.78 | 12.44 | 13.98 | 13.30 | 11.73 |
| Total: | 100.00 | 100.00 | 100.00 | 100.00 | 100.00 | 100.00 |

**S.3.3 Wt% of rGO-T-C(10)**

| Element | Spectrum 1 | Spectrum 2 | Spectrum 3 | Spectrum 4 | Spectrum 5 | Average |
| --- | --- | --- | --- | --- | --- | --- |
| C | 80.97 | 77.25 | 80.02 | 88.14 | 75.02 | 80.28 |
| N | 5.71 | 10.08 | 8.26 | 3.88 | 9.04 | 7.39 |
| O | 13.32 | 12.67 | 11.72 | 7.98 | 15.94 | 12.33 |
| Total: | 100.00 | 100.00 | 100.00 | 100.00 | 100.00 | 100.00 |

**S.3.4 Wt% of rGO-T-C(12)**

| Element | Spectrum 1 | Spectrum 2 | Spectrum 3 | Spectrum 4 | Spectrum 5 | Average |
| --- | --- | --- | --- | --- | --- | --- |
| C | 83.76 | 82.39 | 82.75 | 82.21 | 84.63 | 83.15 |
| N | 4.99 | 6.38 | 7.19 | 7.68 | 5.52 | 6.35 |
| O | 11.25 | 11.23 | 10.06 | 10.11 | 9.85 | 10.50 |
| Total: | 100.00 | 100.00 | 100.00 | 100.00 | 100.00 | 100.00 |
